# Editorial: Using Technology to Combat Diseases and Help People With Disabilities

**DOI:** 10.3389/fpsyg.2022.854762

**Published:** 2022-03-14

**Authors:** Francisco José García-Peñalvo, Henriëtte Geralde Van der Roest, Giovanni Ottoboni

**Affiliations:** ^1^Grupo de Investigación GRIAL, Departamento de Informática y Automática, Instituto Universitario de Ciencias de la Educación, Universidad de Salamanca, Salamanca, Spain; ^2^Trimbos Institute, Utrecht, Netherlands; ^3^Department of Psychology, University of Bologna, Bologna, Italy

**Keywords:** technological ecosystem, digital transformation, people with disabilities, virtual environment, eHealth, user experience, Elderly People, Dementia Care

Nowadays, we live in a technological society that is involved in an unstoppable digital transformation affecting all the business domains, such as education (Castro Benavides et al., 2020; García-Peñalvo, 2021a), industry (Ghobakhloo and Iranmanesh, 2021), tourism (Infante-Moro et al., 2020; Cuomo et al., 2021) or health (Kraus et al., 2021; Kwon et al., 2021; Massaro, in press).

COVID-19 pandemic has accelerated the digital transformation in all the sectors (Cabero-Almenara and Llorente-Cejudo, 2020; Agostino et al., 2021; Argüelles et al., 2021; García-Peñalvo, 2021b; García-Peñalvo et al., 2021a; Hai et al., 2021), but also this disease, beyond its terrible effects, has caused many psychological sequelae both to the patients themselves and to the people who have been confined, but which have been greatly emphasized in people with some type of disability (Akintunde et al., 2021; Wilson et al., 2021; Panchal et al., in press) or mental disorders (Chirico et al., 2021; Ottoboni et al., 2021).

eHealth ecosystems have a significant presence and importance in the current medical practice to create novelty treatments for people who suffer some disabilities or diseases (Marcos-Pablos and García-Peñalvo, 2019; García-Peñalvo et al., 2021b). Telemedicine, powerful surgery assistants, artificial intelligence agents for diagnosis are real examples that we can find in our hospitals for improving the cognitive capabilities of individuals with Down Syndrome, combating nutrition disorders, assisting people with dementia, improving our older people's well-aging, or connecting persons with a physical impediment among other applications.

This Research Topic comprises 11 papers with studies and experiences of using technology for improving the quality of life of people with disabilities or who suffer from diseases, with more interest in the effect of technology than in the technology itself.

*Aesthetically Designing Video-Call Technology with Care Home Residents: A Focus Group Study* (Zamir et al.). This study conducts focus groups with an embedded activity with older people to allow for a person-centered design of a video-call intervention.

*Digital Fabrication and Theater: Developing Social Skills in Young Adults with Autism Spectrum Disorder* (Poveda and Montoya). This article focuses on the digital fabrication workshops, where a group of 10 young individuals with autism spectrum disorder worked on the fundamentals of electronics and programming, as well as 3D design and printing, to make props that were later used on stage in the theatrical performances in which they participated.

*Vocational Training in Virtual Environments for People with Neurodevelopmental Disorders: A Systematic Review* (Michalski et al.). The findings from eight selected studies demonstrate that people with neurodevelopmental disorders can transfer vocational skills from virtual environments to real-world settings.

*Technological Ecosystems that Support People with Disabilities: Multiple Case Studies* (Ramirez-Montoy et al.). This study empirically analyzes the usefulness of treatments that have been supported by technology to answer the question “How do technological ecosystems being used help people with special educational needs?”.

*Knowledge Gaps in Mobile Health Research for Promoting Physical Activity in Adults with Autism Spectrum Disorder* (Lee). This paper provides information on physical activity research and the prospective role of mobile health applications for promoting it in adults with autism spectrum disorder.

*Using Technology to Identify Children with Autism through Motor Abnormalities* (Simeoli et al.). The authors propose using a smart tablet device with a touch screen sensor to capture detailed information about the motor patterns of children with autism spectrum disorder.

*Usability and User Experience of Cognitive Intervention Technologies for Elderly People with MCI or Dementia: A Systematic Review* (Contreras-Somoza et al.). This review finds evidence about usability and user experience measurements and features of stimulation, training, and cognitive rehabilitation technologies for older adults with mild cognitive impairment or dementia.

*Framework for the Classification of Emotions in People with Visual Disabilities through Brain Signals* (López-Hernández et al.). The authors present a 2-fold framework focused on people with visual disabilities that can classify positive and negative emotions.

*Assistive Technologies in Dementia Care: An Updated Analysis of the Literature* (Pappadà et al.). The evidence shows that technology is well-accepted and can support people with dementia and caregivers to bypass physical and environmental problems both during regular times and during future pandemic waves.

*Psychometric Properties of the Spanish Version of Psychosocial Impact of Assistive Devices Scale in a Large Sample of People with Neuromuscular, Neurological, or Hearing Disabilities* (Díez et al.). This study to evaluate measurement properties of the Spanish version of PIADS scale by means of a dataset obtained from its application to a large sample (*n* = 417) of people with neuromuscular, neurological, or hearing disabilities that used different assistive devices.

*Nurturing Grandchildren with Down Syndrome: A Qualitative Study on Grandparents' Needs Using Digital Tools* (Sánchez Gómez et al.). This study analyzes, from a personal perspective, the situations and needs of grandparents who have grandchildren with Down syndrome.

The contributions of all the selected papers and, by extension, of the Research Topic addressed highlight, in the first place, a problem faced by society when its members, due to age or illness, have limited capacities and require greater attention and care. Secondly, technological advances make it possible to intervene in these problems, although they are not magic solutions but tools in the hands of medical professionals, caregivers, and family members that will help both dependent persons and those who care for them. Artificial intelligence is a field that has just begun to be exploited, and that will indeed allow incredible advances in the care and monitoring of people with needs and support for more accurate diagnoses. For all these reasons, we believe that this Research Topic is only a tiny sample of what is being done and what will be done in the coming years, with the aggravating and stimulus while has meant suffering a worldwide pandemic such as that of COVID-19.

## Author Contributions

All authors listed have made a substantial, direct, and intellectual contribution to the work and approved it for publication.

## Conflict of Interest

The authors declare that the research was conducted in the absence of any commercial or financial relationships that could be construed as a potential conflict of interest.

## Publisher's Note

All claims expressed in this article are solely those of the authors and do not necessarily represent those of their affiliated organizations, or those of the publisher, the editors and the reviewers. Any product that may be evaluated in this article, or claim that may be made by its manufacturer, is not guaranteed or endorsed by the publisher.
